# The serial mediating roles of protecting from risks and digital sharing in the relationship between self-control and the desire for being liked

**DOI:** 10.3389/fpsyg.2026.1923963

**Published:** 2026-09-14

**Authors:** İdris Kara

**Affiliations:** Department of Child Development, Vocational School of Social Sciences, Siirt University, Siirt, Türkiye

**Keywords:** desire for being liked, digital parenting awareness, digital protecting from risks, digital sharing, self-control, sharenting

## Abstract

**Purpose:**

This study investigates the mediating role of digital protecting from risks and digital sharing in the influence of one of the Big Five personality traits, namely, self-control, on the desire for being liked. We examine the role of the desire for being liked in relation to the other Big Five personality traits and digital factors such as digital protecting from risks and digital sharing. In addition, the mediating effects of the digital factors in the relationship between personality traits and the desire for being liked remain to be studied in depth.

**Methods:**

By applying a quantitative correlational design, the current study used a sample of 454 parents of preschool-aged children who were recruited via convenience sampling. We tested the indirect effects among the variables using serial multiple mediation analysis performed with PROCESS Macro v4.2 (Model 6).

**Findings:**

The results obtained show that self-control positively predicted protecting from risks (β = 0.57) and negatively predicted the desire for being liked (β = −0.17).

**Conclusion:**

Self-control significantly predicted the desire for being liked indirectly via protecting from risks (Ind1: β = −0.08), sharing (Ind2: β = −0.06), and the serial effect (Ind3: β = −0.06). Our findings suggest that self-control negatively predicts the desire for being liked both directly and indirectly through protecting from risks and sharing. This shows the important need to also target the motive of social approval in digital parenting interventions.

## Introduction

People's social roles, communication, and identity in the digital age have changed. One of the most obvious reflections of this change is that parenting practices have moved from physical boundaries to the virtual space. Parents' behavior of sharing their children's photos, videos, and personal information with large audiences on social media platforms has been called “sharenting,” which is a combination of the English words “share” and “parenting” (Alemdar and Kahraman, 2023; Ayhan and Öztürk, 2021). Although sharenting is based on functional motivations such as strengthening family bonds, accumulating memories, and seeking social support (Blum-Ross and Livingstone, 2017), there are also complex risks related to violating children's privacy rights, constructing digital identities without their consent, and transforming into a means of satisfying parents' approval-oriented psychological needs (Conti et al., 2024; Tosuntaş and Griffiths, 2024).

With the prevalent adoption of social media, parents have transferred their child-rearing practices to digital platforms and have begun to show a tendency to display their parenting performances on this stage. Children have a permanent digital footprint from birth or even before birth. Social platforms have gone beyond being merely a communication tool for parents; they have become platforms where parents prove they are good parents, apply impression management strategies, and seek social approval (Collett, 2005; Ouvrein and Verswijvel, 2019). Parents' momentary decisions can leave their children facing irreversible consequences such as cyberbullying, digital identity theft, and commercial data exploitation in later life (Ayhan and Öztürk, 2021; Kopecky et al., 2020; Steinberg, 2017).

Social media usage rates are making the study of sharenting a more pressing research issue in Turkey. Turkey is one of the top ranking countries in the world in terms of social media usage. It is known that many parents have active accounts on social media and share content about their children on these platforms, specifically Instagram and Facebook (We Are Social and Meltwater, 2024). Studies conducted in Turkey indicate that parents‘ sharenting behaviors are significantly related to demographic and personality variables (Kiliç et al., 2024; Ögel Balaban, 2022). In short, in order to fully understand the psychological factors influencing this behavior, it is important to examine these factors in the local context as well. Enduring personality traits are among the main factors that determine the extent and limits of parents' behaviors on social media.

One of the broadest and best validated personality theories explaining an individual's general behavioral patterns is the Big Five Personality Model (Costa and McCrae, 1992). Self-control (self-control) describes individuals‘ ability to control impulses, follow rules, see long-term consequences, and manage risks (Tangney et al., 2018; Widiger and Costa, 2013). Research implies that parents with high self-control levels tend to develop a higher level of digital parenting awareness about their children's safety in the digital environment (Supti et al., 2025). However, in addition to cognitive risk perception, strong emotional and social motivations can affect parents' sharing behaviors in the digital world.

One of the most significant among them is the desire for being liked. Social media, based on “like” and comments, constantly stimulates people's need for social approval. Sociometer theory (Leary et al., 1995) states that people see virtual likes as real signs of their social acceptability, impacting their momentary self-esteem levels (Burrow and Rainone, 2017). In this sense, parents can present the child as an extension of themselves (extended self) and confirm their parenting identity by sharing their child online (Holiday et al., 2020). This is one of the fundamental dynamics of the “privacy paradox” (Chalklen and Anderson, 2017) that leads parents to share despite knowing the risks.

A review of the literature illustrates that there are few studies that focus structurally on the cognitive and behavioral mechanisms through which parents' core personality traits predict the desire for being liked. It has not been possible to find in the literature any serial mediation model explaining how self-control affects protecting from risks as a cognitive protective mechanism on the one hand and sharing behavior (sharing) on the other, and how it impacts the desire for being liked through these paths. Filling this gap would not only offer a theoretical contribution but could also be useful to inform the development of digital awareness programs for parents.

## Self-control and the desire for being liked

The ability to control one's own actions according to personal values, long-term goals, and disciplines is known as self-control (Tangney et al., 2018). According to Muraven and Baumeister (2000), self-control is a psychological capability that has a restricted amount, becomes exhausted with use, and may be restored with rest and training. People with high self-control capacity can focus on long-term goals rather than seeking instant satisfaction, and they have a stronger attitude toward momentary appreciation and reinforcement signals from the surroundings. Self-control shields a person's self-worth from being dependent on outside sources and anchors it in internal standards (Tangney et al., 2018). The connection between the wish to be admired and low self-control is supported by strong theoretical and empirical evidence.

Collecting likes fast on social media activates the person's reward mechanism quickly, giving short-term mental pleasure. Individuals with low self-control are more vulnerable to quick external gratification and provide a foundation for activities to be guided by the environment's reinforcements rather than the inner value system (Muraven and Baumeister, 2000). In reality, according to a study performed by Seidman (2013), persons who continuously search for approval on social media had low self-control and were highly neurotic. Research conducted by Uziel and Schmidt-Barad (2023) illustrates that the inclination toward impression management has an inversely proportional connection with self-control, implying that those with high self-control capacity can control their urge to manage outside impressions through their inside discipline.

The findings of the research on problematic social media use confirm these findings. A study by Andreassen et al. (2017) on a large-scale national sample showed that addictive level use of social media was significantly related to low self-control. The lower the score for self-control, the higher the chances of getting trapped in the instant approval loops that social media offers. Research on cyber behaviors has shown that self-control can have a determinant effect on FOMO (fear of missing out) and problematic social media use (Supti et al., 2025). Considering this relationship in the context of parenting, parents with high self-control will not feel the need to validate their parenting identity using superficial metrics on social media. Being able to maintain their parental self-efficacy and self-esteem independent from approval signals from their environment, the desire for being liked is expected to lose its power as a behavioral motive. Parents with low self-control could be more susceptible to the structure of social media, offering quick and easy psychological gratification, and prone to acting on the desire for being liked. All things considered, a significant negative relationship between self-control and the desire for being liked is predicted.

### Digital protecting from risks and digital sharing as mediating variables

Beyond the effect on emotional motives, self-control can also have a determining role in cognitive and protective processes. Self-control (Costa and McCrae, 1992) is an internal regulation process that allows an individual to postpone their current desires in order to achieve long-term goals, to make systematic assessments about possible risks, and to act responsibly (Tangney et al., 2018; Widiger and Costa, 2013). Applied to the parenting field, self-control shows as a proactive and protective attitude toward a child's protection in the digital environment. Protecting one's children from risks in the digital environment includes restrictive or active mediation strategies used by parents to shield their children from digital dangers such as cyberbullying, exposure to inappropriate content, digital abuse, and privacy invasions (Livingstone and Helsper, 2008; Symons et al., 2017). Such strategies are not merely technical control strategies but also imply the parents' attention and sensibility toward their child's protection in the digital world. The association between self-control and protecting from risks is based on the fundamental principles of self-control theory.

People with high self-control are more likely to make systematic evaluations about the possible consequences of their decisions, be aware of risky situations, and prepare plans to protect themselves against these situations. These individuals can apply this tendency to the digital area because it is an important factor in predicting health-related protective behaviors (Bogg and Roberts, 2004). A person with high self-control will tend to evaluate the risks that sharing on social media may pose for their child's future digital identity and take protective behaviors accordingly (Dabiriyan Tehrani et al., 2024). This finding is supported by studies conducted by Supti et al. (2025), who found that self-control predicted online safety attitudes better than any other variable. Likewise, Glatz et al. (2018) illustrated that self-control was positively related to actively monitoring children's internet use. As self-control increases, people become more aware of the risks associated with the digital environment and implement protection from risky behaviors more efficiently.

Sharenting is parents' behavior of sharing their children's visual content, videos, and personal information on social media platforms. Although it covers parents' motivations such as seeking social support, accumulating memories, and identity validation, it includes risks that can endanger children's digital security (Blum-Ross and Livingstone, 2017; Steinberg, 2017). It is a logical expectation that parents who have digital risk awareness will restrain themselves to share less. However, research illustrates that parents who worry about digital risks continue to share actively. The concept of “privacy paradox” is used by Chalklen and Anderson (2017) to explain the current contradiction between parents' desire for privacy and approval. Parents with high risk awareness transform their sharing behavior instead of quitting it. Avoiding content that directly reveals the child's identity, preferring posts without location information, and restricting privacy settings to close circles are all signs of the transition (Conti et al., 2024; Walrave et al., 2023; Tosuntaş and Griffiths, 2024). Sharing behavior is not only determined by cognitive risk assessment, but emotional and social motives are also important in determining sharing behavior. Based on sociometer theory (Leary et al., 1995), people change their self-esteem according to the acceptance and rejection signals they receive from their social environment, and likes, comments, and shares on social media platforms are the materialized forms of signals received from the digital environment (Burrow and Rainone, 2017).

In the parenting context, this is related to the concept of impression management (Collett, 2005; Ouvrein and Verswijvel, 2019), where parents build their “good parent” image in the public space by posting about their children and request external validation of this image. Holiday et al. (2020) underlined the fact that parents consider the child as an extension of themselves, and the likes received by the child will help validate the parents' identity. It has been reported that likes and comments are creating a motivational loop to reinforce sharing behavior (Ouvrein and Verswijvel, 2019), specifically in parents with low self-esteem or narcissistic tendencies (Mehdizadeh, 2010; Choi and Lewallen, 2018). The desire for being liked can be considered as a strong motivational variable positively predicting sharing behavior in this context. When all these relationships are assessed together, protecting from risks and sharenting behavior (sharing) can be considered as two different and complementary mediating mechanisms between self-control and desire for being liked.

At the cognitive level, the increase in the parent's self-control ability results in a shift toward protecting the child's digital security (protecting from risks awareness) (self-control → protecting from risks → desire for being liked). At the behavioral level, when the parent is controlling his/her tendency to share less frequently, this also limits the desire for being liked (self-control → sharing → desire for being liked). Apart from these two mechanisms, there is also the serial mechanism (protecting from risks awareness impacts sharing behavior which, in turn, will influence desire for being liked) (self-control → protecting from risks → sharing → desire for being liked). These three indirect pathways are working in a complementary way to constitute the total indirect effect of self-control on the desire for being liked.

### The current study

The current study focuses on the Big Five Personality Model (Costa and McCrae, 1992), self-regulation theory (Muraven and Baumeister, 2000), and sociometer theory (Leary et al., 1995). The desire for being liked is a complex phenomenon that involves the parent's cognitive evaluation processes (self-control and protecting from risks) and sharenting behavior (sharing), which represents the materialization of the social approval motive in the digital environment. Each of these variables has been studied separately but it is of great importance to test all psychological mechanisms in a broad manner and at the same time model the cognitive and behavioral pathways that lead to the desire for being liked. Studies conducted to date have focused on the prevalence of sharenting (Nieto-Sobrino et al., 2025), parental motivations and the privacy paradox (Chalklen and Anderson, 2017; Blum-Ross and Livingstone, 2017), or adolescents' reactions to this phenomenon (Ouvrein and Verswijvel, 2019). We were unable to find any research that has tested, based on a multiple mediation model, the cognitive and behavioral pathways through which personality traits predict the desire for being liked.

There are no holistic models in the literature proposing that self-control (1) strengthens protecting from risks as a cognitive protective mechanism, (2) this protective attitude influences sharenting behavior, and (3) these cognitive and behavioral pathways simultaneously explain three different indirect effects (parallel and serial) leading to the desire for being liked. We test a serial-multiple mediation model including three indirect effects: (a) self-control → protecting from risks → desire for being liked, (b) self-control → sharing → desire for being liked, and (c) self-control → protecting from risks → sharing → desire for being liked. The participants of this study are parents. In Turkey, only a few studies were conducted on sharenting and these studies showed that sharenting behaviors of parents are related to some demographic and psychological factors (Kiliç et al., 2024; Ögel Balaban, 2022). In addition to its contribution to the literature of Turkey, since the intensity of social media use in Turkey is high (We Are Social and Meltwater, 2024), investigating the personality roots of sharenting of Turkish parents can create the opportunity to create data that can be used as a basis for comparison in countries with similar cultures.

The findings to be obtained will (1) contribute theoretically to the field by indicating that sharenting behavior is not just about using technology, but also about managing deep personality and psychological needs, (2) serve as an evidence-based guide on what kind of psychological capacity needs to be improved when developing digital awareness programs for parents, and (3) add to policy discussions aimed at protecting children's privacy rights in the digital environment. In line with all this information, the aim of the study is to examine the mediating role of digital protecting from risks and digital sharing in the relationship between self-control and the desire for being liked.

**H1:** Self-control significantly predicts the desire for being liked in a negative direction.

**H2:** Digital protecting from risks has a mediating effect in the relationship between self-control and the desire for being liked.

**H3:** Digital protecting from risks and digital sharing have a serial-multiple mediation effect in the relationship between self-control and the desire for being liked.

Note: Consistent with regression-based mediation terminology (Hayes, 2018), the terms “predicts,” “effect,” and “mediation” are used throughout this manuscript in their statistical (associational) sense, as conventionally used within the PROCESS framework, and do not imply causal precedence, given the cross-sectional design of the present study. Findings are more precisely described as indirect associations rather than causal mediation effects.

## Method

### Research design

This is a predictive correlational study that aims to reveal the relationship between self-control, protecting from risks, sharenting behavior (sharing), and the Desire for being liked, as well as the indirect effects between them. The type of study allows identifying the relationships between two or more variables and the direct and indirect effects within these relationships (Büyüköztürk et al., 2017). SPSS 26.0 (IBM Corp, 2019) was used for data analysis. Serial multiple mediation analysis was conducted with PROCESS Macro v4.2 (Model 6) developed by Hayes (2018) with 5,000 bootstrap resamples. The evaluation of the fit of the models was based on 95% bootstrap confidence intervals.

## Participants

### Study sample and demographic characteristics

This study was conducted on 454 participants consisting of social media users selected through convenience sampling. Demographic information regarding the participants is presented in Table 1.

**Table 1:** Demographic information of participants.

Variable	Category	*n*	%
Age	20–25 years	12	2.6
	26–31 years	143	31.5
	32–37 years	175	38.5
	38–43 years	93	20.5
	44 years and above	31	6.8
	**Total**	**454**	**100.0**
Gender	Female	345	76.0
	Male	109	24.0
	**Total**	**454**	**100.0**
Education level	Primary school	40	8.8
	Secondary school	48	10.6
	High school	106	23.3
	University	215	47.4
	Postgraduate	45	9.9
	**Total**	**454**	**100.0**
Income level	Low	94	20.6
	Medium	76	16.6
	Good	57	12.4
	Very Good	227	50.3
	**Total**	**454**	**100.0**
Employment status	Unemployed	222	49.0
	Employed	221	48.8
	Student	11	2.2
	**Total**	**454**	**100.0**
Social media usage duration	Less than one h	148	32.6
	2–4 h	276	60.8
	5–8 h	26	5.7
	More than 8 h	4	0.9
	**Total**	**454**	**100.0**
Social media privacy	Public	44	9.7
	Private	122	26.9
	Visible to acquaintances only	288	63.4
	**Total**	**454**	**100.0**

Table 1 shows that the largest group in terms of age distribution was the 32–37 age range at 38.5% (*n* = 175). This was followed by the 26–31 age range at 31.5% (*n* = 143) and the 38–43 age range at 20.5% (*n* = 93). Participants aged 44 and above constituted 6.8% (*n* = 31) of the sample. Meanwhile, those in the 20–25 age range constituted 2.6% (*n* = 12). In terms of gender, the majority of participants were female (76.0%; *n* = 345), while male participants made up only 24.0% (*n* = 109) of the sample. Regarding education level, approximately half of the sample had a university degree (47.4%; *n* = 215). High school graduates ranked second at 23.3% (*n* = 106), while postgraduate degree holders constituted 9.9% (*n* = 45), secondary school graduates 10.6% (*n* = 48), and primary school graduates 8.8% (*n* = 40). In terms of income level, over half of the participants (50.3%; *n* = 227) rated their income as “very good.” Those at the “low” income level constituted 20.6% (*n* = 94), those at the “medium” level 16.6% (*n* = 76), and those at the “good” level 12.4% (*n* = 57).

Regarding employment status, participants were distributed approximately equally between unemployed (49.0%; *n* = 222) and employed (48.8%; *n* = 221) groups. Students constituted 2.2% (*n* = 11) of the sample. In terms of social media usage duration, most participants used social media for 2–4 h daily (60.8%; *n* = 276). Those who used it for less than one h constituted 32.6% (*n* = 148), those using 5–8 h 5.7% (*n* = 26), and those using it over 8 h 0.9% (*n* = 4). Finally, regarding social media privacy preferences, the majority of participants (63.4%; *n* = 288) kept their profiles “visible to acquaintances only.” Those who preferred “private” profiles constituted 26.9% (*n* = 122), while those who preferred “public” constituted 9.7% (*n* = 44).

### Statistical power analysis of sample size

A priori power analysis was performed with G^*^Power 3.1 (Faul et al., 2009) to evaluate the sample size in terms of statistical power for the mediation model. The PROCESS-based serial multiple mediation analysis applied in the study is fundamentally based on multivariate linear regression equations (Hayes, 2018). Therefore, power calculations were performed with the F test for linear multiple regression (Fixed model, *R*^2^ deviation from zero).

Based on the observed *R*^2^ = 0.36 in the third regression equation, the most complex equation in the model (Dependent variable: Desire for being liked; Predictors: Self-control, Protecting from Risks, Sharing + Age and Gender as control variables; k = 5), the effect size was calculated as f^2^ = *R*^2^/(1 − *R*^2^) = 0.36/0.64 = 0.5625 (large effect). This effect size (f^2^ = 0.56), under the conditions of α = 0.05 and 1 − β = 0.80, shows the minimum required sample size for k = 5 predictors is *N* = 25. Even with a more conservative approach that assumes a medium effect size (f^2^ = 0.15), the required minimum sample is *N* = 92. The current sample (*N* = 454) exceeds these thresholds.

Fritz and MacKinnon (2007) simulation-based reference table was examined to assess the statistical power required for detecting indirect effects in bootstrap-based mediation analyses. About *N* = 200–250 participants are sufficient to achieve 80% power with 5,000 bootstrap resamples for small-to-medium indirect effect sizes (a × b ≈0.14). Even though the indirect effect sizes observed in the study (Ind1: β = −0.08; Ind2: β = −0.06; Ind3: β = −0.06) are small, the sample of *N* = 454 provides statistical power exceeding 99% for effects of this magnitude. The sample size was assessed to meet sufficient statistical power for individual regression equations and bootstrap mediation figures.

## Data collection tools

### Personal information form

A personal information form was prepared by the researcher to collect participants' demographic information. Additionally, four different measurement instruments were used: Digital Sharing Scale, Digital Protecting from Risks Scale, Desire for being liked Scale, and Self-control Scale. All scales used had been previously developed with established Turkish validity and reliability studies.

### Digital sharing scale

The Sharenting Scale was prepared by Alemdar and Kahraman (2023). The scale has a total of 17 items. The scale includes 4 sub-dimensions, including sharing behavior (6 items), social comparison behavior (4 items), positive feedback effect (4 items), and negative feedback effect (3 items). The scale includes 1-6 items for sharing behavior, 14-17 items for social comparison behavior, 7-10 items for positive feedback effect, and 11-13 items for negative feedback effect. The scale has a 5-point Likert-type scale (1 = Strongly disagree, 5 = Strongly agree). Items included in the social comparison behavior and negative feedback effect sub-dimensions are reverse coded. Higher scores obtained from the scale indicate higher sharenting behavior. The Cronbach's alpha internal consistency coefficient of the scale was reported as α = 0.73. In this study, the sharing behavior sub-dimension was used to measure digital sharing. Reliability analysis of the Digital Sharing Scale indicated that Cronbach's alpha coefficient was 0.91 and McDonald's omega coefficient was 0.91. An example of an item included in the scale is “I find it appropriate to share posts (photos/videos/texts) about my child on social media” (Alemdar and Kahraman, 2023).

### Digital protecting from risks scale

Manap and Durmuş (2020) designed the Digital Parenting Awareness Scale, which includes 16 items in total. It has four sub-dimensions, namely: Protecting from Risks (PfR), Efficient Use (EU), Being a Negative Role Model (BNRM), and Digital Neglect (DN). There are 4 items in each sub-dimension. The scale is measured using a 5-point Likert-type scale (1 = Never, 5 = Always). The score of each sub-dimension ranges between 4 and 20. Scores obtained from the Protecting from risks and Efficient Use sub-dimensions are higher for individuals who have high digital parenting awareness. Scores obtained from the Being a Negative Role Model and Digital Neglect sub-dimensions are higher for individuals who have low digital parenting awareness. Cronbach's alpha coefficients of BNRM, DN, EU, and PfR were found to be 0.79, 0.78, 0.71, and 0.63, respectively. Reliability analysis suggested that Cronbach's alpha coefficient of the Protecting from Risks sub-dimension was 0.88 and McDonald's omega coefficient was 0.88 in the current sample. This value is notably higher than the α = 0.63 originally reported for this sub-dimension by Manap and Durmuş (2020). Such a discrepancy may reflect sample-specific characteristics, item functioning differences across samples, or contextual factors, and should not be interpreted as sufficient evidence of construct validity on its own. Because internal consistency alone cannot confirm that the sub-dimension functions as intended in the current sample, this discrepancy is discussed further as a limitation of the study (see Limitations). A sample item from the scale is “I am able to protect my child from the risks of the internet” (Manap and Durmuş, 2020).

### Desire for being liked scale

The desire for being liked Scale was developed by Kaşikara and Dogan (2017) and is a unidimensional measurement tool consisting of 9 items. The scale aims to measure individuals' desire for being liked and approved in social settings. A 4-point Likert-type rating is used (1 = Strongly disagree, 4 = Strongly agree). There are no reverse-coded items. Higher scores indicate a higher desire for being liked. The Cronbach's alpha internal consistency coefficient was reported as α = 0.81. Reliability analysis yielded a Cronbach's alpha of 0.81 and a McDonald's omega of 0.81. A sample item is “Thinking that I will not be liked worries me” (Kaşikara and Dogan, 2017).

### Self-control scale

In this study, the self-control sub-dimension of the 10-item Big Five Personality Scale developed by Rammstedt and John (2007) and adapted into Turkish by Horzum et al. (2017) was used to measure individuals' personality traits. The scale measures five fundamental personality dimensions: extraversion, agreeableness, self-control, neuroticism, and openness to experience. The Turkish adaptation study confirmed that the internal consistency and composite reliability values of the scale were at adequate levels. Accordingly, for extraversion internal consistency was 0.88 and composite reliability 0.83; for agreeableness 0.81 and 0.73; for self-control 0.90 and 0.85; for neuroticism 0.85 and 0.79; and for openness to experience 0.84 and 0.78. In this study, the self-control dimension was used to measure self-control, and the Cronbach's alpha coefficient was found to be 0.78. A sample item is “I see myself as someone who tends to be slow to act” (Horzum et al., 2017).

It should be noted that the self-control dimension of the Turkish BFI-10 adaptation is measured with only two items. While this brevity is practical for large-scale survey administration and is consistent with the original short-form design of the instrument (Rammstedt and John, 2007), it restricts the construct's bandwidth and may not fully capture the multidimensional nature of self-control as conceptualized by Tangney et al. (2018), which encompasses impulse control, healthy habits, work ethic, and reliability facets. The self-control sub-dimension was nonetheless selected for this study because it directly corresponds to the Big Five facet most theoretically relevant to self-regulation processes implicated in sharenting behavior (Muraven and Baumeister, 2000). The reliability coefficient obtained in the current sample (α = 0.78) should be interpreted with some caution given this brevity, as internal consistency estimates from two-item scales can be less stable than those from longer instruments and are strongly determined by the correlation between the two items themselves. This limitation is discussed further below.

## Procedure

The study was approved by the Siirt University Social Research Ethics Committee (Date: 05.04.2024, reference number: 6770). All stages of the study were conducted in accordance with the Declaration of Helsinki. Data were collected using a Google Form. A form was sent to participants through social media tools. The first part of the form consisted of informed consent. The informed consent form contained information on the aim of the study, its approximate duration, and participants' rights such as their right to withdraw from the study if they wanted to do so. All participants who voluntarily participated in the study gave their consent. The inclusion criterion was having a preschool-aged child. The criterion was confirmed through participants' self-reports. No identifying information was collected to ensure the anonymity and confidentiality of the responses. Participants were also asked not to write any personal information.

## Data analysis

IBM SPSS Statistics 26.0 was used to analyze the data. Pearson correlation analysis was used to examine the relationships between research variables. The Model 6 serial multiple mediation model of PROCESS Macro v4.2 was used to determine the direct and indirect effects. Indirect effects were considered significant considering the 95% confidence intervals calculated with 5,000 bootstrap samples. When the bootstrap confidence intervals do not contain the zero value, the indirect effect is statistically significant. Age and gender variables were taken into account as control variables in all analyses. Before testing the relationships among variables in the research model and the mediating variable effects, the data were examined for univariate and multivariate outliers. For this purpose, standardized Z scores were first examined to detect univariate outliers. Five data points that were found to be outliers based on the accepted ±3.29 threshold in the literature were removed from the dataset. Multivariate outliers were detected by using Mahalanobis distance values. The significance level (df = 4, *p* < 0.001) and critical chi-square value (χ2 = 18.47) were used as the basis.

Three observations that exceeded this threshold were removed from the dataset. The values of VIF (risk = 1.394; sharing = 1.304; self-control = 1.177) are much lower than the value of 5, while the values of tolerance (risk = 0.717; sharing = 0.767; self-control = 0.850) are greater than the value of 0.10. In order to check whether the errors in the model are independent of each other, the Durbin-Watson coefficient is calculated for testing independence of error terms. This value has been found to be within the accepted value range (DW = 1.787). Linearity, homoscedasticity, and normality of residuals were not formally tested in the present analysis; this omission is acknowledged as a methodological limitation (see Limitations), and the regression-based and indirect effects reported below should be interpreted with this caveat in mind. Means, standard deviations, skewness, and kurtosis values of the variables and relations between variables were also examined. As all variables were taken at the same time from the same subjects via self-reporting, there was a risk of common method bias (CMB) (Podsakoff et al., 2003). The risk of CMB was investigated by using Harman's single-factor test. When all variables were placed on one factor in an exploratory factor analysis, the percentage of total variance explained by this factor was 36.4%. Since this ratio is far less than the generally accepted 50%, it can be said that there was no serious common method bias.

## Findings

The relationships among variables were examined using Pearson correlation coefficients, and the results are presented in Table 2.

**Table 2:** The relationships between variables.

Variables	1	2	3	4
1 = Desire for Being Liked	1			
2 = Self-control	−0.34^*^	1		
3 = Protecting from risks	−0.43^*^	0.37^*^	1	
4 = Sharing	0.54^*^	−0.28^*^	−0.47^*^	1
Mean	2.70	3.61	2.66	3.34
Std. deviation	0.858	0.802	1.21	1.24
Skewness	−0.654	0.042	0.310	−0.631
Kurtosis	−0.446	−0.190	−1.05	−0.741

^*^p < 0.01.

As shown in Table 2, the relationships among variables were examined using Pearson correlation analysis. According to the results, the desire for being liked was negatively and significantly associated with self-control (r = −0.34, *p* < 0.05) and protecting from risks (r = −0.43, *p* < 0.05), and positively and significantly associated with sharing (r = 0.54, *p* < 0.05). Additionally, self-control was positively associated with protecting from risks (r = 0.37, *p* < 0.05) and negatively associated with sharing (r = −0.28, *p* < 0.05). The protecting from risks variable had a negative and significant relationship with sharing (*r* = −0.47, *p* < 0.05). Skewness and kurtosis values indicate that the data are approximately normally distributed.

## Mediation analysis

In the scope of the mediation analysis, the mediating role of the protecting from risks and sharing variables in the effect of self-control on the desire for being liked was tested using the mediation model. The results are presented in Table 3 (see Figure 1).

**Table 3:** Mediational model coefficients.

Predictors	Protecting from risks		Sharing		Desire for being liked	
	β	*p*	β	*p*	β	*p*
Self-control	0.57	0.001	−0.21	0.001	−0.17	0.001
Protecting from risks	-	-	−0.42	0.005	−0.13	0.001
Sharing	-	-	-	-	0.28	0.001
Constant	0.25	0.43	6.05	0.001	2.62	0.005
	*R* = 0.38, *R*^2^ = 0.14		*R* = 0.52, *R*^2^ =0.27		*R* = 0.59; *R*^2^= 0.36	
	*F*_(3, 450)_ = 25.14, *p* < 0.001		*F*_(4, 449)_ = 41.27, *p* < 0.001		*F*_(5, 448)_ = 49.7, *p* < 0.001	
	**Estimates of Point** β		**The lowest**		**The highest**	
Total	−0.200		−0.267		−0.142	
Ind1	−0.08		−0.124		−0.033	
Ind2	−0.06		−0.108		−0.018	
Ind3	−0.06		−0.096		−0.042	

Ind1 = Self-control → Protecting from risks → Desire for being liked.

Ind2 = Self-control → Sharing → Desire for being liked.

Ind3 = Self-control → Protecting from risks → Sharing → Desire for being liked.

**Figure 1:**
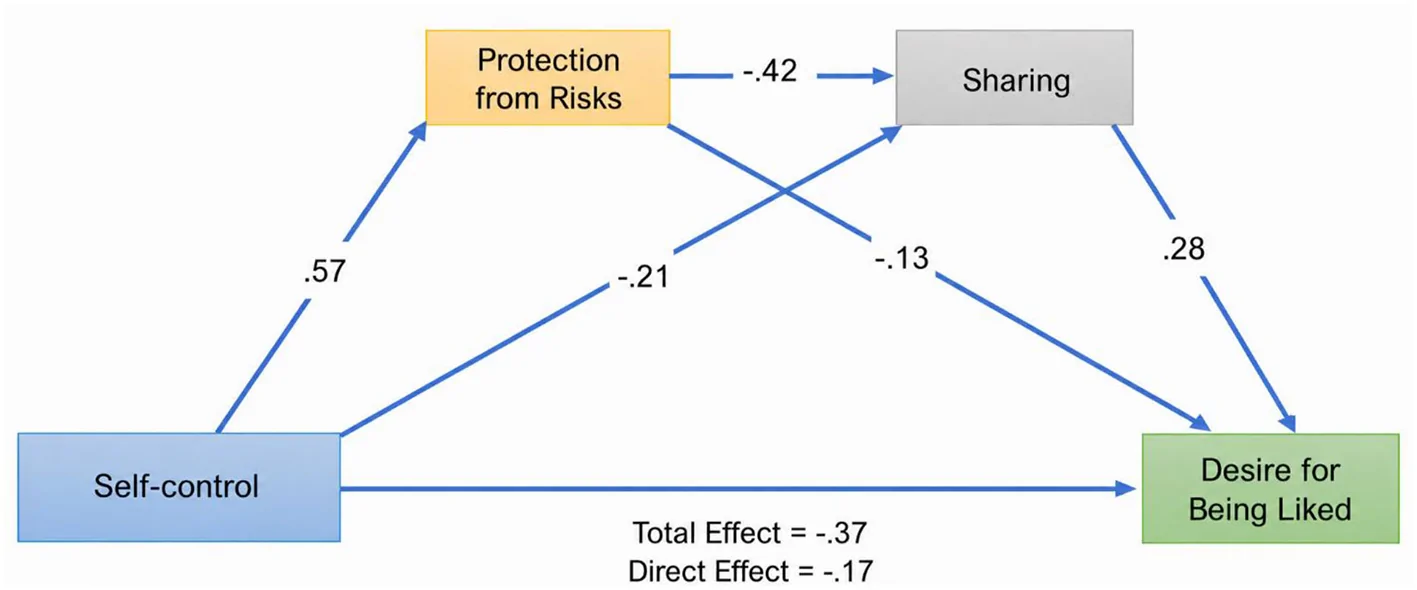
The chain mediation role of protecting from risks and sharing in the effect of self-control on the desire for being liked. Age and gender variables were controlled for all dependent and mediating variables in the model.

In the first model, self-control had a positive and significant effect on protecting from risks (β = 0.57; *p* < 0.001). In the second model, self-control (β = −0.21; *p* < 0.001) and protecting from risks (β = −0.42; *p* = 0.005) negatively predicted the sharing variable at a significant level. In the third model, self-control (β = −0.17; *p* < 0.001) and protecting from risks (β = −0.13; *p* < 0.001) negatively predicted the desire for being liked, while the sharing variable positively predicted it (β = 0.28; *p* < 0.001). When model results are examined, the explained variance in the model where self-control predicted protecting from risks was *R*^2^ = 0.14 [*F*_(3, 450)_ = 25.14, *p* < 0.001]. The explained variance in the model where self-control and protecting from risks predicted that sharing was *R*^2^ = 0.27 [*F*_(4, 449)_ = 41.27, *p* < 0.001]. The explained variance in the model where self-control, protecting from risks, and sharing jointly predicted the desire for being liked was determined to be *R*^2^ = 0.36 [*F*_(5, 448)_ = 49.71, *p* < 0.001]. The explained variance in the model examining only the total effect of self-control on the desire for being liked was *R*^2^ = 0.13 [*F*_(3, 450)_ = 21.77, *p* < 0.001].

The bootstrap results from the mediation analysis show the total indirect effect of self-control on the desire for being liked is significant [β = −0.200; 95% CI (−0.267; −0.142)]. When specific indirect effects are examined, all three indirect effects were significant. The indirect effect of self-control on the desire for being liked through protecting from risks [β = −0.08; 95% CI (−0.124; −0.033)], through sharing [β = −0.06; 95% CI (−0.108; −0.018)], and also through the sequential effect of protecting from risks and sharing [β = −0.06; 95% CI (−0.096; −0.042)] were significant.

The regression coefficients are also presented in the table. The total effect of self-control on the desire for being liked was significant at β = −0.37 [SE = 0.047; *p* < 0.001; 95% CI (−0.461; −0.276)]. The direct effect was β = −0.17 [SE = 0.044; *p* < 0.001; 95% CI (−0.255; −0.081)].

The results of the bootstrap analysis, based on 5,000 resamplings, are shown in Table 4. The findings also support the proposed chain mediation model. The direct effect of self-control on the desire for being liked is negative and significant [β = −0.17; 95% CI (−0.254; −0.087)]. The effect of self-control on protecting from risks is positive and significant [β = 0.57; 95% CI (0.436; 0.697)]. Meanwhile, its effect on sharing is negative and significant [β = −0.21; 95% CI (−0.342; −0.076)]. The effect of protecting from risks on sharing was observed to be negative and significant [β = −0.42; 95% CI (−0.505; −0.330)]. Protecting from risks negatively and significantly predicted the desire for being liked [β = −0.13; 95% CI (−0.194; −0.070)]. The sharing variable had a positive and significant effect on the desire for being liked [β = 0.28; 95% CI (0.221; 0.342)]. These findings show that the effect of self-control on the desire for being liked occurs through direct and indirect pathways via the protecting from risks and sharing variables, and that the chain mediation structure proposed in the model is supported.

**Table 4:** Results of bootstrapping analyses for the chain mediation model.

Model pathways	Coefficient	Confidence interval (%95)	
		Lower	Upper
a → b	−0.17	−0.254^*^	−0.087^*^
a → c	0.57	0.436^*^	0.697^*^
a → d	−0.21	−0.342^*^	−0.076^*^
c → d	−0.42	−0.505^*^	−0.330^*^
c → b	−0.13	−0.194^*^	−0.070^*^
d → b	0.28	0.221^*^	0.342^*^

a: self-control, b: desire for being liked, c: protecting from risks, d: sharing. ^*^indicates that the 95% bootstrap confidence interval does not include zero (statistically significant path).

## Discussion

The findings of this study confirm all three hypotheses tested (H1, H2, and H3). In addition to illustrating that parents' desire to gain social approval in the digital space is not a simple cause-and-effect process but a multi-layered and complex process where personality traits, risk perception, and parenting behavior intertwine (Tosuntaş and Griffiths, 2024), the structural findings obtained reveal that “protecting from risks” and “sharenting behavior” (sharing) mediate the effects of parents' self-control levels on the desire for being liked. It can be concluded from these findings that parents who have high self-control levels are less concerned about being validated by the parenting identity they project on social media using its superficial like metrics and the desire for being liked loses its meaning as a behavioral motive for these parents. Findings on the first hypothesis (H1) reveal that self-control directly predicts the desire for being liked in a statistically significant negative way (β = −0.17, *p* < 0.001).

These results support the related literature in the field. Self-regulation theory (Muraven and Baumeister, 2000) indicates that individuals with high self-control capacity base their self-worth on themselves (internal standards, long-term goals, personal values) instead of seeking it from other people and external sources. They are less likely to fall into the trap of instant external rewards offered via social media's “like” and comment functions (Tangney et al., 2018). Research done by Seidman (2013) showed that individuals who constantly sought social media's approval scored low in terms of self-control. In addition, Uziel and Schmidt-Barad (2023) proved that the desire to manage one's impression is negatively related to self-control. Andreassen et al. (2017) indicated, in a large sample study, that the probability of falling into the instant approval cycles offered by social media platforms decreased as the score of self-control increased.

Confirmation of the second hypothesis (H2) suggests that the “digital protecting from risks” variable also has a significant mediating effect on the relationship between self-control and the desire for being liked [Ind1: β = −0.08, 95% CI (−0.124; −0.033)]. Considering the values of path coefficients, self-control positively predicts protecting from risks (β = 0.57), while protecting from risks negatively predicts the desire for being liked (β = −0.13). This suggests that parents with high self-control capacity have protective attitudes toward the child's digital safety (Glatz et al., 2018; Supti et al., 2025), and this protective orientation in parenting was associated with a lower desire for being liked. A parent with motivation to protect their child from digital risks determines their parenting performance not with the use of external reinforcement, but with the sense of responsibility of ensuring the safety of their child. It is the same type of parenting attitude defined in the literature as “child-focused digital parenting,” which stands in opposition to the social approval motive (Nikken and Jansz, 2014; Livingstone et al., 2017).

Regarding the negative association between protecting from risks and sharing (β = −0.42), one plausible explanation is that heightened risk awareness does not necessarily lead parents to abandon sharing altogether, but rather to adopt more selective and restrained sharing practices. Consistent with the privacy paradox literature (Chalklen and Anderson, 2017; Walrave et al., 2023), risk-aware parents may increasingly filter the content they share-for instance, by avoiding face-revealing images or restricting audience visibility-which, within the scope of the sharing behavior sub-dimension used in this study, is reflected as reduced sharing frequency rather than complete cessation.

Protecting from risks awareness represents a type of cognitive buffer that was associated with a reduced need for external approval in parents with high self-control. The result brings a new dimension to the discussion of the privacy paradox by showing that the protection motive, to some extent, was associated with a lower desire for being liked: risk awareness does not always prevent the sharing decision, but it is related to a reduced need for social approval in the parent (Chalklen and Anderson, 2017; Tosuntaş and Griffiths, 2024). Confirmation of our third and most broad hypothesis (H3) reveals the serial-multiple mediation effect of the “protecting from risks” and “sharing” variables in the relationship between self-control and the desire for being liked [Ind3: β = −0.06, 95% CI (−0.096; −0.042)]. The formation of parents' social approval motive follows a serial chain consisting of different steps: self-control → protecting from risks → sharing → desire for being liked (along the personality-cognition-behavior axis). First, parents with higher self-control will make great efforts to protect their children from digital threats. Such a protective attitude (protecting from risks) is associated with a more restrained frequency of sharing, and this reduction in sharing behavior is, in turn, associated with less content receiving likes and comments, corresponding with a weaker social approval cycle.

Second, this structural mechanism tends to show that the social reinforcement cycle feeding the desire for being liked in parents (i.e., more sharing leads to more likes, and more likes lead to a stronger desire for being liked [Ouvrein and Verswijvel, 2019]) can be broken by self-control and risk awareness. Sociometer theory posits that people regulate their self-esteem according to the acceptance signals provided by their environment (Leary et al., 1995). Self-control partially disables this system and reframes the need for social approval in the parent in terms of internalized standards (Burrow and Rainone, 2017; Sherman et al., 2016). Self-control appears to be associated with a cognitive-behavioral regulation sequence in which risk awareness (protecting from risks) is linked to sharing behavior, which in turn is associated with a lower desire for being liked (Ranzini and Lutz, 2016). Our findings show that the desire for being liked is not simply a personality weakness but is associated with a systematic psychosocial process involving personality traits, risk perception, and sharing behavior. Self-control shows a negative association with the desire for being liked both directly (via self-worth independent of external approval) and indirectly through protecting from risks and reduced sharing behavior. This makes a unique contribution to the literature.

The relatively strong correlation between sharing and the desire for being liked (r = 0.54) merits closer conceptual scrutiny, as it represents one of the strongest associations observed in the model. One interpretation is that sharing functions instrumentally as a strategy through which parents actively seek social validation, consistent with impression-management accounts of sharenting (Collett, 2005; Ouvrein and Verswijvel, 2019). At the same time, some conceptual overlap between the sharing behavior sub-dimension and the desire for being liked construct cannot be ruled out, as both instruments partly tap into approval-oriented motivations. Future research could examine the discriminant validity between these two constructs, for example through confirmatory factor analysis, to clarify whether they represent empirically distinct phenomena.

## Limitations and future perspectives

The present study has several limitations. The number of items used to measure self-control is limited (two items). The BFI-10 self-control scale reflects each personality dimension with only 2 items, which limits the accuracy of measuring such a complex concept. This measurement brevity may have compressed the observed effect sizes and the explained variance in models involving self-control (e.g., *R*^2^ = 0.14 in the first regression equation), potentially attenuating the true strength of the associations tested. Future studies should consider using longer, multi-item self-control measures—such as the Brief Self-Control Scale (Tangney et al., 2004)—to obtain more robust and construct-valid estimates. Secondly, the ratio of female participants is high (76%) and this restricts the generalization of results to the whole parent population and hinders us from completely exploring the existing gender differences in sharenting. Thirdly, the use of convenience sampling restricts the representativeness of the sample with respect to the Turkish parent population. Fourthly, the cross-sectional research design prevents making causal inferences and provides no information about how relationships between variables change over time. It should be noted that although the term “mediation” is used throughout the manuscript for consistency with the PROCESS framework (Hayes, 2018), the cross-sectional nature of the data does not allow for establishing temporal precedence or causality among the variables. Therefore, the findings should be interpreted as “indirect effects” rather than true mediation in the causal sense. Hayes and Rockwood (2017) have emphasized that cross-sectional mediation models can identify indirect effects, but longitudinal or experimental designs are necessary to confirm causal mediation pathways.

A further methodological limitation concerns the absence of a comprehensive assessment of regression-specific assumptions-linearity, homoscedasticity, and normality of residuals-beyond the multicollinearity (VIF, tolerance) and autocorrelation (Durbin-Watson) diagnostics reported in the Method section. Future replications of this model should report a complete set of regression diagnostics to strengthen confidence in the regression-based mediation estimates. Consequently, the regression-based path coefficients and indirect (mediation) effects reported in this study should be interpreted with appropriate caution, as violations of these assumptions, if present, could affect the precision of the standard errors and confidence intervals obtained.

Another limitation concerns the reliance on internal consistency coefficients alone as evidence for the adequacy of the measurement instruments in this sample. In particular, the considerably higher alpha obtained for the Protecting from Risks sub-dimension (α = 0.88) compared to the original validation study (α = 0.63) suggests that additional construct validity evidence, such as confirmatory factor analysis, would strengthen confidence in the instrument's functioning within this population. Future research should incorporate such analyses rather than relying exclusively on reliability estimates. No additional construct validity analyses (e.g., confirmatory factor analysis or other validity indicators) were conducted in the present study beyond the reliability estimates reported above, primarily due to constraints of the current dataset; we acknowledge this as a limitation to be addressed in future replications using this instrument. Relatedly, the use of isolated sub-dimensions from broader instruments (the Protecting from Risks sub-dimension of the Digital Parenting Awareness Scale, and the sharing behavior sub-dimension of the Sharenting Scale) requires justification: these sub-dimensions were selected because they most directly correspond to the cognitive and behavioral constructs of theoretical interest in the present model, but their use in isolation from the broader instruments they belong to may not fully capture the multidimensional nature of digital parenting awareness or sharenting behavior more broadly.

The income distribution anomaly is apparent in that 50.3% of participants stated their income level as “very good,” which is not in line with the Turkish average. Thus, there might be some selection bias in the convenience sample. Another limitation is common method bias. We collected all variables at the same time from the same participants via self-report. Although we conducted a Harman's single-factor test to control this common method bias, it should be noted that this test, although widely used, is now considered a relatively weak safeguard against common method bias, as it can fail to detect method variance in more complex models (Podsakoff et al., 2003). Because all constructs were measured via self-report at a single time point, shared method variance may have inflated some of the observed associations, particularly the correlation between sharing and the desire for being liked. Future studies should consider more rigorous designs, such as multi-trait multi-method approaches, time-lagged data collection, or the inclusion of a marker variable, to more adequately assess and control for common method bias. It is impossible to fully eliminate self-report method bias in a design such as this one. In future research, multi-source data collection methods (e.g., observational data or platform usage statistics) should be used. Social desirability bias is a related limitation: parenting behavior and social approval motive self-reports might be affected by participants' tendency to answer in socially desirable ways, thus limiting their validity specifically for socially sensitive variables like the desire for being liked (Fisher, 1993). Another limitation is that our sample consists solely of parents with preschool-aged children. Since the child's age and developmental level can differentiate sharenting behaviors, the generalizability of results to parents of school-aged or adolescent children should be considered carefully.

Finally, only age and gender were included as control variables in the present models. Other potentially relevant factors-such as the frequency and intensity of social media use, platform type, parental digital literacy, child characteristics, and prior experience with online risks-were not controlled for and may confound or moderate the associations reported here. Future studies should incorporate a broader set of control variables to more precisely isolate the associations of interest.

## Conclusions and recommendations

The results of this study showed that the desire for being liked -parents' need for social approval on the internet-is not solely a matter of personality weakness or careless use of technology. It is the result of a multi-layered, complex process in which the parents' self-control, the protective awareness created against digital risks, and sharenting behavior are intertwined (Tosuntaş and Griffiths, 2024). The research model testing the hypotheses empirically illustrated that self-control was negatively associated with the desire for being liked, both directly and indirectly, through protecting from risks and sharing (Tangney et al., 2018; Muraven and Baumeister, 2000). This structural chain of associations offers a useful framework for interpreting the “privacy paradox” (privacy-openness paradox) often debated in the literature on digital parenting in relation to fundamental personality characteristics (Chalklen and Anderson, 2017). Parents with high levels of self-control and self-regulation have the potential to foresee and handle risks that their children may face in the online environment, such as cyberbullying, inappropriate content, and digital footprint (Nikken and Jansz, 2014). They think that they can neutralize threats by using the privacy options provided by digital platforms or filtering techniques like hiding their children's faces effectively and feel safe within the borders of “mindful sharenting” (Walrave et al., 2023; Cino and Wartella, 2021). However, despite all these actions taken to protect digital privacy, the desire for being liked is not completely eliminated in parents.

Even high risk aware parents tend to place their children in their extended self and request recognition of their parenting skills (Collett, 2005; Holiday et al., 2020). This means that they need social approval for being a good parent. The study found that high self-control is associated with greater protection-from-risk awareness, which in turn is associated with less frequent sharing behavior; less frequent sharing, in turn, is associated with a weaker “like” cycle and a lower desire for being liked. The study suggests that self-control is associated with a lower desire for being liked both directly (through the internal value system) and indirectly (through its association with protecting from risks and sharing) (Ranzini and Lutz, 2016; Tosuntaş and Griffiths, 2024). When creating their digital identity, parents may underestimate their children's privacy rights, future safety, and yet-to-be expressed digital consent (Kopecky et al., 2020; Steinberg, 2017). The findings of the current study show that it is possible to address this situation not only by increasing risk awareness but also by designing psychological interventions targeting the social approval motive feeding the desire for being liked. Strengthening self-control may be associated with greater protective attitudes and could plausibly weaken the desire for being liked as a behavioral driver; however, longitudinal or experimental designs would be needed to test this possibility directly.

## Recommendations

When sharing content about their children on social media, parents should recognize the influence of the desire for likes on their decision-making processes and should prioritize the irreversible effects of such sharing on the child's future digital footprint and psychological integrity (Kopecky et al., 2020; Conti et al., 2024). Specifically, avoiding content that directly reveals the child's identity, restricting privacy settings to close circles, and obtaining sharing consent from children who have reached a certain developmental level (Sarkadi et al., 2020) can be adopted as concrete indicators of self-control-oriented mindful parenting practices (Walrave et al., 2023).

We also provide some recommendations for practitioners and policy makers. To protect children's rights in the digital space, it would be useful to organize psycho-educational programs to increase parents‘ digital literacy and privacy awareness. Based on the results of the current study, it seems that simply increasing parents' risk awareness may not be enough to completely suppress their desire for being liked. Therefore, the contents of such programs should also include strengthening self-control skills and addressing the need for belonging and competence behind the external approval motive. Combining self-regulation-focused intervention programs (Muraven and Baumeister, 2000) with mindfulness-based approaches (Williams-Ceci et al., 2021) can be an effective way to achieve this goal.

It will be important for future research to test the structural model established in this study (based on self-control, protecting from risks, sharing behavior, and the desire for being liked) using longitudinal designs to better understand how the relationships among these variables develop over time as the child grows (Tosuntaş and Griffiths, 2024). Another important future research direction would be mixed-methods research that focuses on how adolescents perceive their parents' sharing motivations and desire for being liked (Verswijvel et al., 2019). Because the current study is conducted in the Turkish context, it should be interpreted in a culture-specific way. Conducting comparative research across different cultural contexts to understand how collectivist cultural structure affects the relationship between social approval motive and self-control (Hofstede, 2001) is one of the most important priorities for future research.

## Data Availability

The raw data supporting the conclusions of this article will be made available by the authors, without undue reservation.
